# Designing a Virtual Reality Café to Support People with Eating Disorders

**DOI:** 10.1192/bjo.2026.11227

**Published:** 2026-06-30

**Authors:** Laura Chapman, Ian Penton-Voak, Lucy Yardley, Helen Bould

**Affiliations:** 1 University of Bristol, Bristol, United Kingdom; 2 Gloucestershire Health and Care NHS Foundation Trust, Gloucester, United Kingdom

## Abstract

**Aims:**

People with eating disorders can find the experience of social eating difficult, and being able to eat socially can be an important part of recovery. In the context of mental health treatment, experiences of mastery within virtual reality (VR) can be transferred to real life, and previous qualitative research has identified that a VR café environment could be a useful treatment adjunct for people with eating disorders. The aim of this project was to develop a VR café intervention for people with eating disorders who find social eating challenging.

**Methods:**

We used the Person-Based Approach to develop the VR café. In Study 1 we conducted qualitative interviews and focus groups with young people with personal experience of a range of different eating disorders (n*=*15), parents and carers (n*=*4), and clinicians from a variety of professional backgrounds (n*=*6), to inform the design of the intervention. In Study 2, the café was further developed through a series of development activities and think aloud interviews with people with experience of eating disorders (n*=*12).

**Results:**

Study 1 participants described a range of challenges associated with cafés, which were incorporated into the initial build of the VR café by our industry partners,*Virtual Bodyworks (Kiin).* These included challenges related to social interactions with café staff, the busyness of café settings, and around choosing and ordering food and drink from café menus. Study 2 participants further shaped the intervention, contributing to the scripts for VR café staff; helping to develop food and drink menus; providing feedback on measures for assessing the effectiveness of the intervention; and advising on the ways clinicians could helpfully support people using the VR café, including through the provision of feedback on a written guidance document. During think-aloud interviews, Study 2 participants also identified areas for improvement to the content and experience of the VR café.

**Conclusion:**

By placing the perspectives and ideas of people with eating disorders at the heart of the design of the VR café, we have developed a safe space for people with diverse eating disorders to practice relevant challenges related to social eating whilst being supported by a clinician. The intervention aims to reduce anxiety, increase confidence, and improve tolerance of uncertainty around social eating settings, and will be offered to young people accessing eating disorder treatment in two participating NHS trusts, as part of a mixed methods feasibility trial.

